# Supplementary material for: Imaging the field inside nanophotonic accelerators
Source: Nat Commun. 2023 Jun 21;14:3687. doi: 10.1038/s41467-023-38857-z (PMC10284904; doi:10.1038/s41467-023-38857-z)
Supplement: Supplementary file 1 — Supplementary Information [file 41467_2023_38857_MOESM1_ESM.pdf]

Supplementary Information:
Imaging the field inside nanophotonic accelerators

Tal Fishman[1,†], Urs Haeusler[2,3,†], Raphael Dahan[1], Michael Yannai[1], Yuval Adiv[1], Tom Lenkiewicz Abudi[1], Roy Shiloh[2], Ori Eyal[1], Peyman Yousefi[2], Gadi Eisenstein[1], Peter Hommelhoff[2], Ido Kaminer[1]

[†] These authors contributed equally to this work.

[1] Department of Electrical and Computer Engineering, Technion – Israel Institute of Technology, Haifa 32000, Israel
[2] Department of Physics, Friedrich-Alexander-Universität Erlangen-Nürnberg (FAU), Staudtstraße 1, Erlangen 91058, Germany
[3] Cavendish Laboratory, University of Cambridge, JJ Thomson Avenue, Cambridge CB3 0HE, UK

Email: ftal@technion.ac.il, kaminer@technion.ac.il

1 Experimental Setup

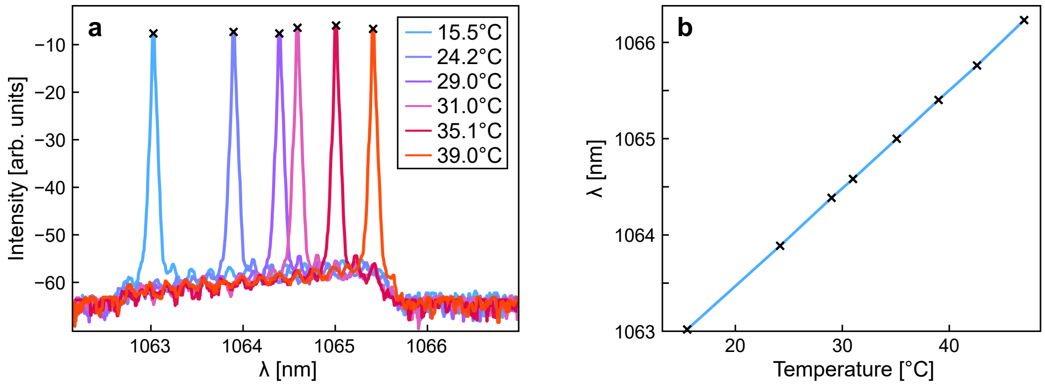

Supplementary Figure S1. Measured laser spectrum vs. thermo-electric cooler temperature. **(a)** Spectra at various temperatures. **(b)** Peak wavelength vs. temperature extracted from (a). The blue solid line is a fit that confirms the linear scaling.

2 Photonic inverse design and 3D simulations

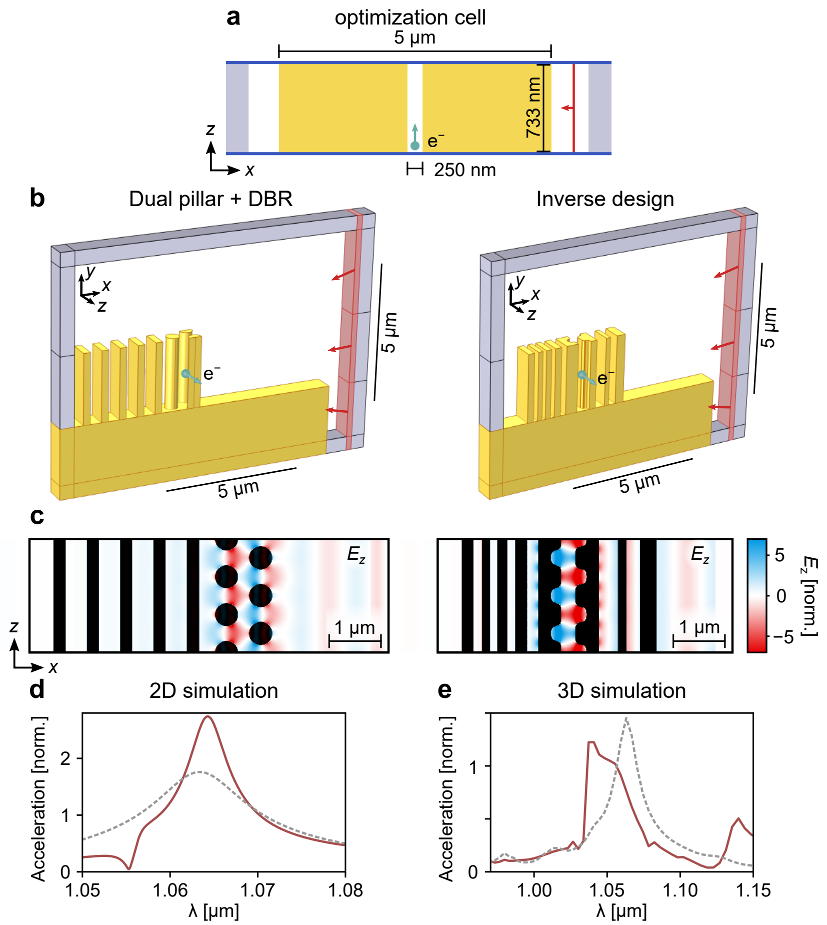

Supplementary Figure S2. Comparison between 2D and 3D simulations. (a) Two-dimensional simulation cell as used for the inverse-design optimization and the spectral response (d). A single unit cell of the grating is optimized. Periodic boundaries (blue) along the longitudinal direction are applied to simulate an infinitely long grating with a periodicity of 733 nm. The design region (yellow) is 5 µm wide and includes a 250-nm-broad vacuum channel (white) in the middle. A plane wave (red) is impinging from the right-hand side. Perfectly matched layers (gray) are used to absorb light and imitate free space. **(b)** The simulation cells for the 3D-FDFD simulations of the dual-pillar (left) and inverse-designed (right) structures. The 2.5-µm-tall nanostructure sits on a mesa and a Gaussian beam is focused onto the center of the electron channel. **(c)** Electric field distribution $E_z(x,z)$ for the dual-pillar (left) and inverse-designed (right) structures, obtained by 2D simulation. **(d, e)** Calculated spectral response of the dual-pillar (dashed, gray line) and inverse-designed (solid, red line) structure from 2D and 3D simulation, respectively. While the 2D simulation (d) predicts a stronger acceleration gradient and a narrower spectral response for the inverse-designed structure, the two structures perform similarly in 3D (e).

We used an open-source Python package based on a 2D-FDFD simulation (Fig. S2a) [1]. The structure was optimized over a 5-µm-wide design region in the xz-plane, containing a 250-nm-wide vacuum channel in the center for the electrons to propagate through. Periodic boundaries were

applied along the z-direction, enforcing the periodicity of 733 nm, and perfectly matched layers were defined along x. A transverse-magnetic plane wave was excited from one side, and the resulting electric field E_z was computed in the center of the channel to find the acceleration gradient, which served as the objective function of the optimization [2, 3].

3D-FDFD simulations were performed in COMSOL. The simulation cell (Fig. S2b) consists of one unit cell of the structure with periodic boundaries along the z-direction. The 2.5-µm-high structure sits on a mesa, with the electron channel 5.5 µm away from the mesa edge. Perfectly matched layers enclose the simulation cell along the x- and y-direction. A Gaussian beam with a beam waist of 15 µm FWHM is focused onto the center of the channel at a height of 1.8 µm above the mesa. An angle of incidence of 5° above the substrate was observed to reproduce the experimental data best and was therefore chosen.

Figure S2c shows the 2D simulated electric field E_z in the xz-plane for the two structures. Figures S2d and e show the spectral response of the dual-pillar (dashed line) and the inverse-designed (solid line) structures from 2D and 3D simulations, respectively. The 2D simulated spectrum reveals the maximum acceleration gradient at the designed wavelength of 1064 nm. The bandwidth of the inverse-designed structure is twice as narrow as that of the dual-pillar structure, indicating the resonant nature of the former. By contrast, the 3D simulation shows a slightly narrower response of the dual-pillar structure, which is in accordance with the observed narrower cosh-like mode with respect to changes in structure diameter (see Fig. 4 of the main text). The stark difference between the 2D and 3D results are a testimonial to the importance of 3D simulations, and the excellent agreement of our experimental data with the 3D results confirms their accuracy.

Another difference is that the 3D resonance frequencies of the inverse-designed and dual-pillar structures are shifted to 287 THz (1045 nm) and 284 THz (1056 nm). Ultimately, a 3D inverse-design optimization should be performed.

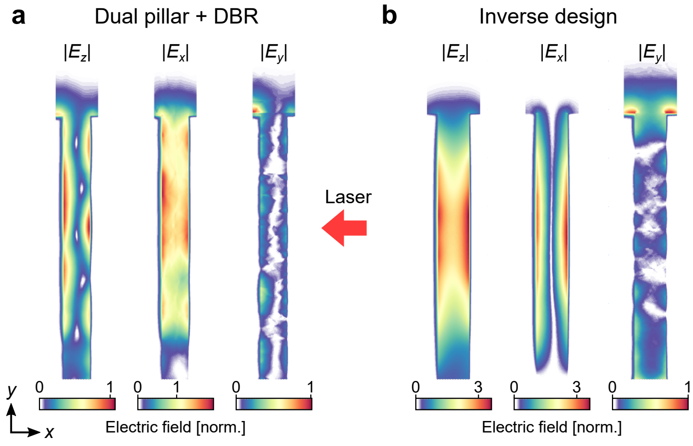

Supplementary Figure S3. Simulated field components inside the DLA channel. The field simulations for the design parameters which best reproduce the experimental field measurement are shown for the **(a)** dual-pillar and **(b)** inverse-designed structure. It demonstrates that an anti-symmetric mode in E_z is accompanied by a symmetric mode in E_x and vice versa. The electric field E_y along the vertical direction is close to zero inside the channel and only strong above the structure.

3 Data processing

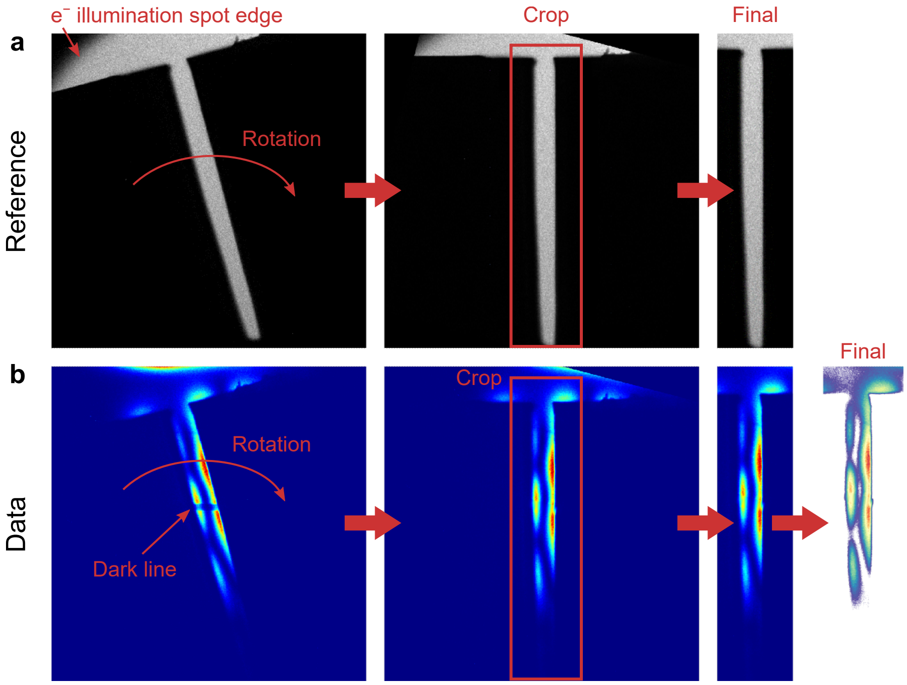

Supplementary Figure S4. Process of data manipulation. (a) Unfiltered image as reference. **(b)** Energy-filtered TEM (EFTEM) image. First, the images are rotated by a bilinear transformation. Second, a dark line artifact in the filtered image is removed by linear extrapolation of the neighboring points. The colormap in (b) gives the false impression of slightly curved channel walls, which is an artifact of having a larger field strength at central height of the channel.

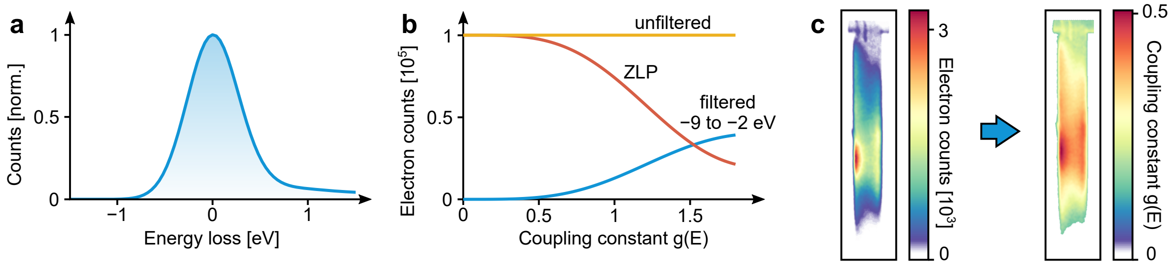

Supplementary Figure S5. Transformation of EFTEM image to g-factor distribution. (a) Fitted electron energy loss spectrum (EELS) of the zero-loss peak (ZLP). The experimentally observed asymmetry in the spectrum was modeled as a sum of a Gaussian and an 11% contribution from an exponentially shifted Gaussian, i.e., the amount by which the Gaussian was shifted to the right-hand side exponentially decays. **(b)** Electron counts as a function of g, calculated via equation (1) of the main text. The yellow line shows the electron counts without energy filtering, i.e., integration of the EELS over the entire energy axis. The unfiltered EFTEM image without laser illumination served as the normalization for our image transformation to a g-factor distribution. The red line shows the ZLP integrated over the range -2 to +2 eV, which strictly speaking includes the ZLP and the first sidebands. The blue line shows the number of electrons that gained energy, i.e., the integration of the EELS from -9 to -2 eV, as used for the EFTEM images. **(c)** Transformation from electron counts (left image) to g factors (right images). It uses the blue line from (b) to map electron counts to g factors. Small electron numbers (below a chosen threshold) cannot be accurately mapped to g factors and were removed.

4 Proposed tomography method

To image the field distribution in subsections of the structure, we suggest to either place an aperture in front of the area of interest or to focus the laser beam onto that region. Figure S5 shows 2D simulations for those two scenarios and confirms that the acceleration profile closely follows the excitation beam shape. As long as the beam is not focused too tightly or the aperture is too small, diffraction effects are minor. In the proposed 3D tomography method, the laser beam is scanned along the structure length, or the aperture is placed at multiple locations along the structure. A deconvolution algorithm is then applied to retrieve the field information as a continuous function of z. For the acceleration of sub-relativistic electrons, the structure periodicity is tuned to maintain synchronicity as the electron velocity increases. To image the field at a farther point along the structure, it is therefore necessary to pre-accelerate the electrons to the velocity of each subsection.

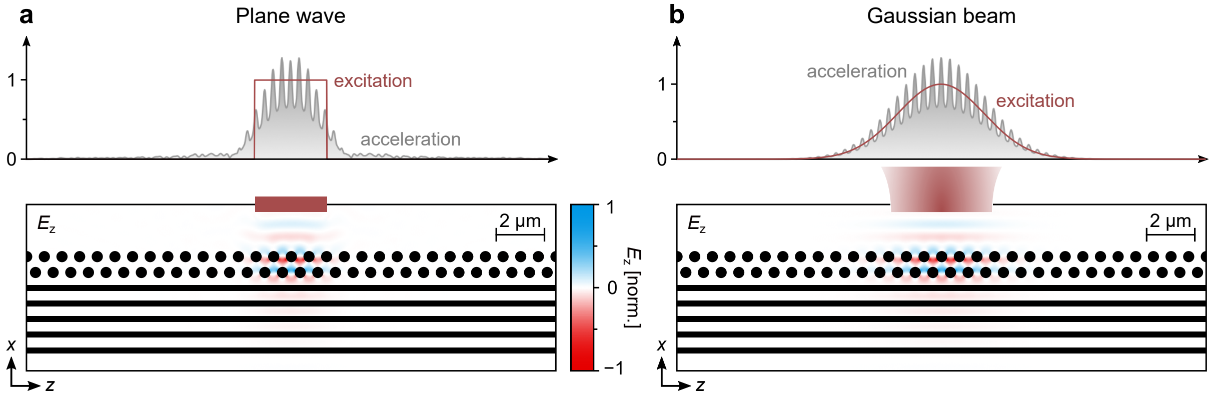

Supplementary Figure S6. Proposed illumination schemes for 3D field tomography. The acceleration profile is shown together with the shape of the excitation laser beam. Underneath is the 2D field simulation in the xz-plane. The two proposed schemes are **(a)** a plane-wave excitation as achieved by placing an aperture in front of the structure, and **(b)** a focused Gaussian beam. In both cases, the acceleration profile closely follows the excitation.

5 Phase matching and Fourier mode sampling

In our experiment, the electron samples the Fourier mode $q = \omega/v_\mathrm{e}$ of the electric field in the z-direction. If we neglect edge effects and assume the structure is uniformly illuminated over its interaction length L, then the Fourier transform of the field inside the structure is that of a square pulse of length L multiplied by a periodic function with structure periodicity Λ. In other words, it is the convolution of the sinc-function $\mathrm{sinc}(qL)$ with a sum over delta-functions at $q = n \cdot 2\pi/\Lambda$, where n is the integer mode number. Here, the electron dominantly couples to the $n = -1$ mode, for which we find the Fourier transform to be $\tilde{E}(q) = \mathrm{sinc}\left[(2\pi/\Lambda - q)L\right]$. This means the Fourier component at the electron sampling frequency $q = \omega/v_\mathrm{e}$ is proportional to $\mathrm{sinc}\left[(2\pi/\Lambda - \omega/v_\mathrm{e})L\right]$. From this, we can retrieve the synchronicity condition $2\pi/\Lambda = \omega/v_\mathrm{e}$, which can be rewritten to $\lambda = \Lambda/\beta$.

The first zero crossing of our signal $\mathrm{sinc}\left[(2\pi/\Lambda - q)L\right]$ with $L = 15\,\mu\mathrm{m}$ occurs for a wavelength of $\lambda = 1064\,\mathrm{nm}$ at an electron velocity of $\beta = 0.672$ $(179\,\mathrm{keV})$ or $\beta = 0.706$ $(211\,\mathrm{keV})$, and for

an electron velocity of $\beta = 0.69$ at a wavelength of $1037\,\mathrm{nm}$ or $1089\,\mathrm{nm}$. This means that for the parameters used in our experiment ($\beta = 0.69$ and $\lambda = 1063 - 1065.4\,\mathrm{nm}$) we do not expect to see a significant drop in signal due to phase mismatch.

Supplementary References

1. Hughes, T. W., Williamson, I. A., Minkov, M. & Fan, S. Forward-mode differentiation of Maxwell's equations. *ACS Photonics* **6,** 3010–3016 (2019).
2. Hughes, T., Veronis, G., Wootton, K. P., England, R. J. & Fan, S. Method for computationally efficient design of dielectric laser accelerator structures. *Optics express* **25,** 15414–15427 (2017).
3. Sapra, N. V. *et al.* On-chip integrated laser-driven particle accelerator. *Science* **367,** 79–83 (2020).
